# Supplementary material for: Intermuscular adipose tissue and muscle function in patients on maintenance hemodialysis
Source: Physiol Rep. 2025 Jul 9;13(13):e70363. doi: 10.14814/phy2.70363 (PMC12241717; doi:10.14814/phy2.70363)
Supplement: Supplementary file 1 — Figure S1. Flow charts of MHD and Control groups, respectively. Figure S2. Comparison of Inflammatory marker concentrations between groups. [file PHY2-13-e70363-s002.docx]

**MHD**


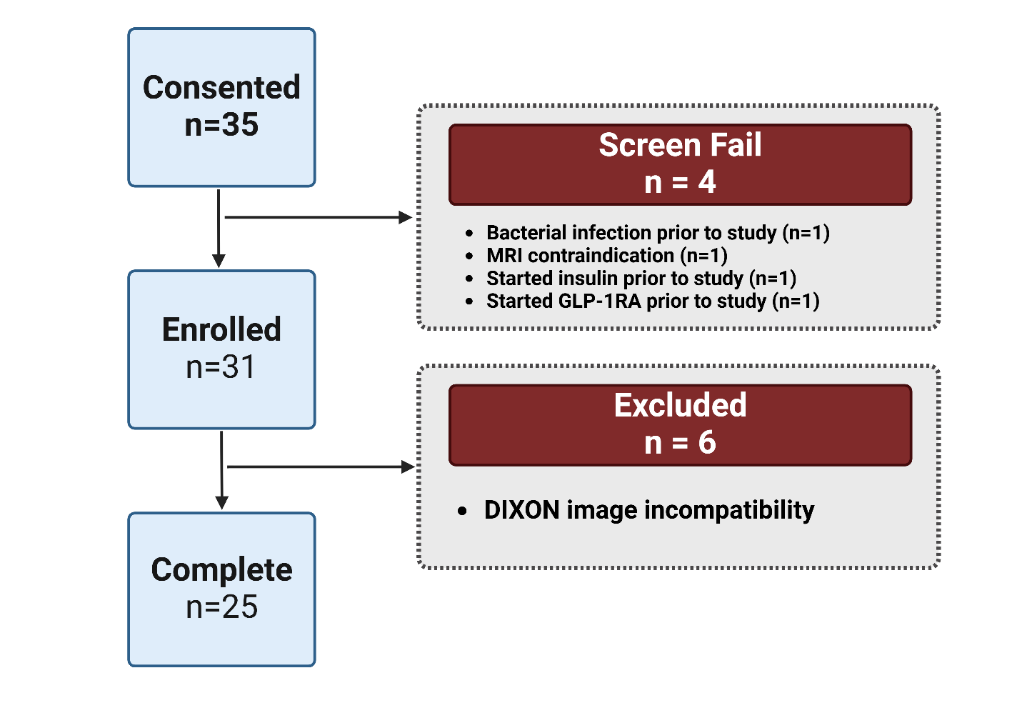


**Control**

**
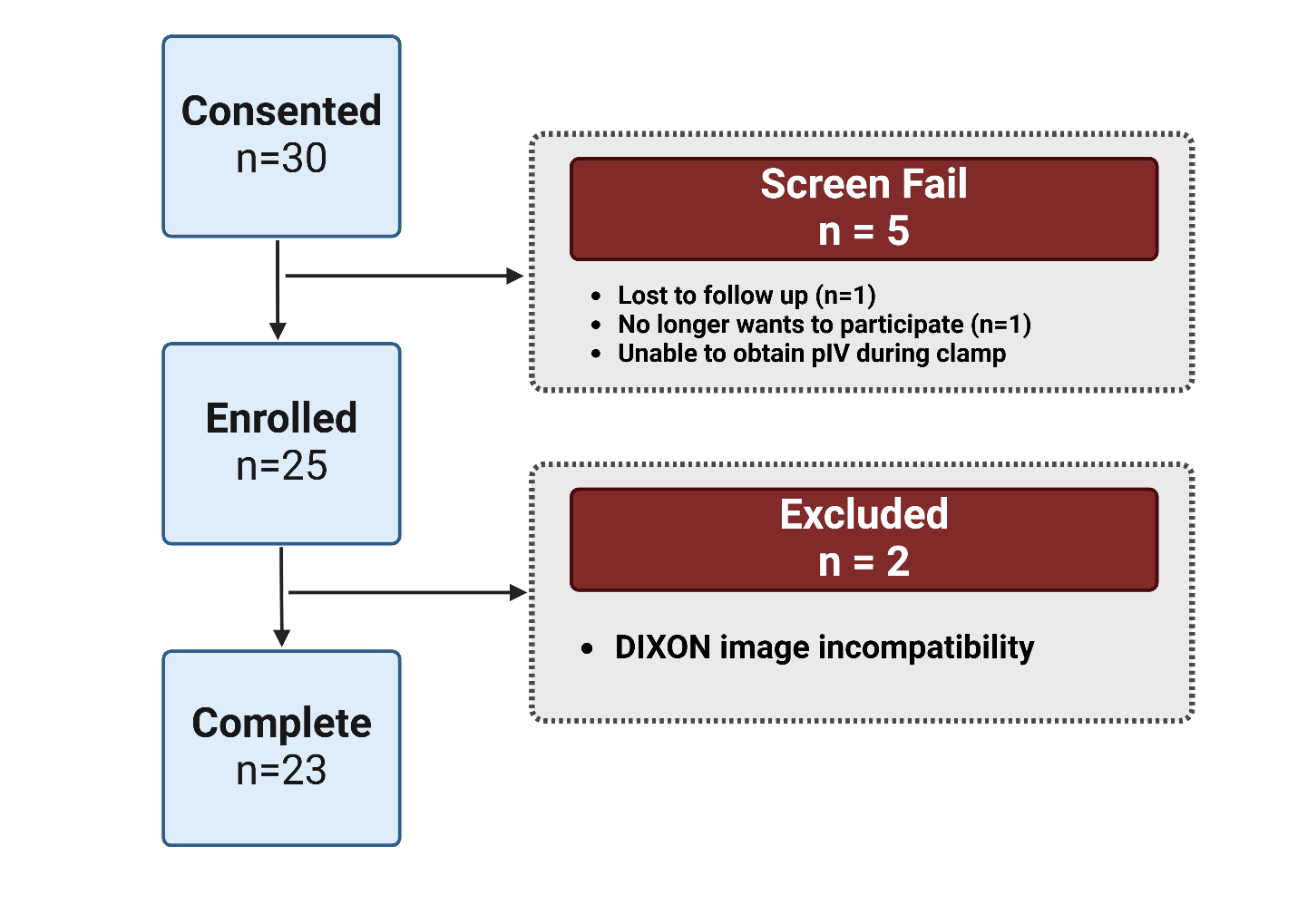
**

***Created with BioRender.com***

**Figure S1. Flow charts of MHD and Control groups respectively**


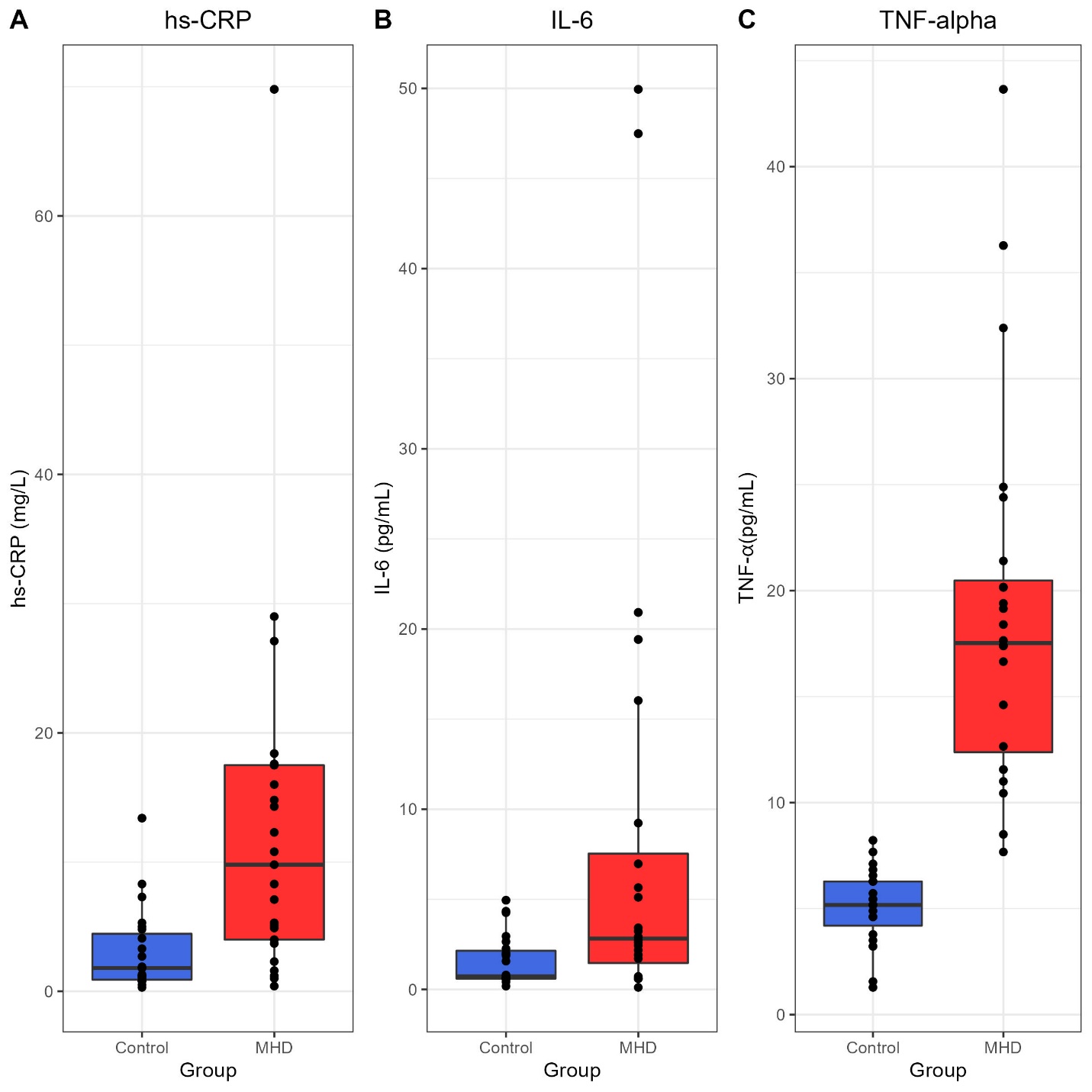


**Figure S2. Comparison of Inflammatory marker concentrations between groups.** **(A), (B), (C)** Box plot for TNF- α, IL-6, and hs-CRP concentrations comparison between control and MHD groups (p < 0.001, p = 0.004, and p < 0.001, respectively.)
